# Preparation, Characterization, and In Vitro Release of Curcumin-Loaded IRMOF-10 Nanoparticles and Investigation of Their Pro-Apoptotic Effects on Human Hepatoma HepG2 Cells

**DOI:** 10.3390/molecules27123940

**Published:** 2022-06-20

**Authors:** Dongge Yin, Xueling Hu, Mengru Cai, Kaixin Wang, Hulinyue Peng, Jie Bai, Yvchen Xv, Tingting Fu, Xiaoxv Dong, Jian Ni, Xingbin Yin

**Affiliations:** School of Chinese Material Medica, Beijing University of Chinese Medicine, Beijing 102488, China; qq1280247000@163.com (D.Y.); hxlhbsy1@163.com (X.H.); 20210941442@bucm.edu.cn (M.C.); wkxsuga924@163.com (K.W.); phlyue@163.com (H.P.); bj19960928@163.com (J.B.); 20200935155@bucm.edu.cn (Y.X.); futt1701@163.com (T.F.)

**Keywords:** MOFs, IRMOF-10, curcumin, HepG2 cells

## Abstract

Curcumin (CUR) has a bright future in the treatment of cancer as a natural active ingredient with great potential. However, curcumin has a low solubility, which limits its clinical application. In this study, IRMOF-10 was created by the direct addition of triethylamine, CUR was loaded into IRMOF-10 using the solvent adsorption method, and the two were characterized using a scanning electron microscope (SEM), X-ray diffraction (XRD), dynamic light scattering (DLS), Fourier transform infrared spectroscopy (FTIR), thermogravimetric analysis (TG) methods, and Brunauer–Emmett–Teller (BET) analysis. We also used the MTT method, 4′,6-diamidino-2-phenylindole (DAPI) staining, the annexin V/PI method, cellular uptake, reactive oxygen species (ROS), and the mitochondrial membrane potential (MMP) to perform a safety analysis and anticancer activity study of IRMOF-10 and CUR@IRMOF-10 on HepG2 cells. Our results showed that CUR@IRMOF-10 had a CUR load of 63.96%, with an obvious slow-release phenomenon. The CUR levels released under different conditions at 60 h were 33.58% (pH 7.4) and 31.86% (pH 5.5). Cell experiments proved that IRMOF-10 was biologically safe and could promote curcumin entering the nucleus, causing a series of reactions, such as an increase in reactive oxygen species and a decrease in the mitochondrial membrane potential, thereby leading to cell apoptosis. In summary, IRMOF-10 is an excellent drug carrier and CUR@IRMOF-10 is an effective anti-liver cancer sustained-release preparation.

## 1. Introduction

**Figure 1:**
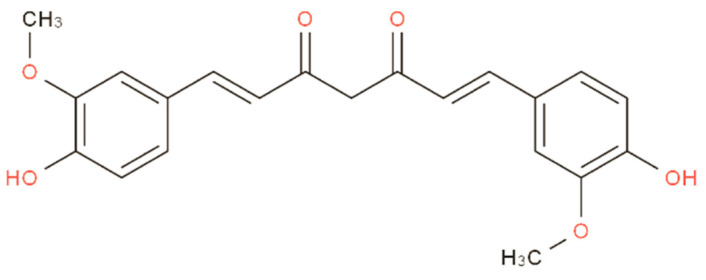
Chemical structure diagram of curcumin.

Curcumin (CUR), a diketone compound extracted from the rhizomes of some plants in the Zingiberaceae and Araceae families, is a natural anticancer and anti-inflammatory active ingredient (Figure 1). It can treat nervous system diseases caused by inflammation or oxidative stress [1,2,3,4]. It can also reduce bone pain caused by cancer [5]. Curcumin-related clinical trials have involved a wide range of diseases, proving its safety for each [6]. Liver cancer is the most common primary liver malignant tumor with high recurrence and high metastasis [7]. Curcumin is effective in treating liver cancer. The main mechanism is to inhibit the proliferation of liver cancer cells and induce autophagy and apoptosis of liver cancer cells, inhibit the metastasis and invasion of liver cancer cells, reverse the drug resistance of liver cancer cells, and inhibit tumor angiogenesis and metastatic growth [8]. Although curcumin has shown excellent prospects in cancer treatment, its clinical application is limited due to its low solubility [9]. To overcome this, researchers have attempted to wrap curcumin with nanomaterials, and have tested various nano preparations, such as liposomes, micelles, nanogels, and nanoemulsions [10].

**Figure 2:**
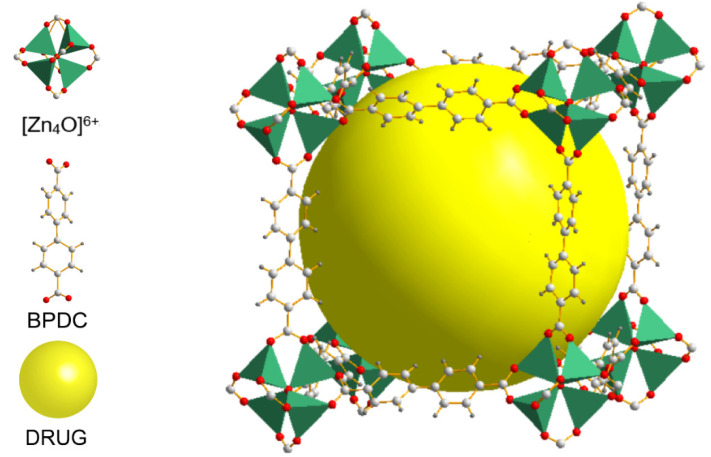
Schematic illustration of the construction of IRMOF-10.

Metal–organic frameworks (MOFs) have the advantages of offering many types: a large specific surface area, adjustable pore size, high stability, and good biocompatibility [11,12,13,14]. The differences in type and pore size influence the drug-loading capacity of the MOFs, as well as their drug-releasing performance. The series of IRMOFs synthesized by Yaghi et al. are all block topologies. The IRMOF series are microporous crystalline Zn_4_O(R1-BDC)_3_ materials comprising [Zn_4_O]^6+^ inorganic groups and a series of aromatic carboxylic acid ligands, which are bridged and self-assembled in the form of octahedrons; the pore sizes are all mesopores in the range of >20 Å [15]. IRMOF-1 is the simplest structure in this series, synthesized from Zn(NO_3_)_2_·4H_2_O and terephthalic acid (H_2_BDC) in *N*,*N’*-diethylamine (DEF) solvent. For IRMOF-1, this series can increase the pore size using longer dicarboxylic acid ligand reactants [16], of which IRMOF-10 is a typical example (Figure 2). IRMOF-10 has a similar structure to IRMOF-1 but is significantly larger. IRMOF-10 was obtained by changing the organic ligand to 4,4′-biphenyldicarbonyl chloride (BPDC) based on IRMOF-1. IRMOF-10 has been documented to have good hydrogen storage capacity [17], CO_2_ adsorption capacity [18,19], and methane adsorption capacity [20]. In addition, researchers have developed various functions for IRMOF-10. John et al. combined IRMOF-10 with the transition metal catalyst gold (III) to reduce the decomposition of gold and render it recyclable, greatly improving the efficiency of heterogeneous catalysis [21]. IRMOFs have good stability, high drug loading, and good biocompatibility characteristics. Therefore, research on IRMOFs as drug carriers has attracted much attention in recent years. Studies have shown that IRMOFs hold tremendous potential for drug loading and release. For example, IRMOF-3 contains norcantharidin (NCTD) used to treat primary liver cancer [22], MOF-5 contains oridonin [23], and IRMOF-74-III contains gemcitabine (GEM) to treat cancer [24].

In this study, we selected IRMOF-10 as the carrier of curcumin, synthesized IRMOF-10 by the direct addition method of triethylamine, carried CUR using the solvent adsorption method, and investigated the in vitro release characteristics of IRMOF-10. The materials were characterized by SEM, XRD, DLS, FTIR, TG, and BET. We investigated the biological safety of IRMOF-10 using an MTT assay, 4′,6-diamidino-2-phenylindole (DAPI) staining, and an annexin V/PI assay with HepG2 cells. Potential experiments were conducted to preliminarily investigate the anti-liver cancer effect of CUR@IRMOF-10, cell uptake, ROS, and the mitochondrial membrane. The safety of IRMOF-10, its drug-release performance, and the enhancement of CUR’s anti-tumor effect are discussed, providing a reference for IRMOF-10 as a drug carrier.

## 2. Results

### 2.1. Synthesis and Characterization

IRMOF-10 was synthesized by the triethylamine method and characterized by SEM, XRD, DLS, FTIR, TG, and BET assay.

**Figure 3:**
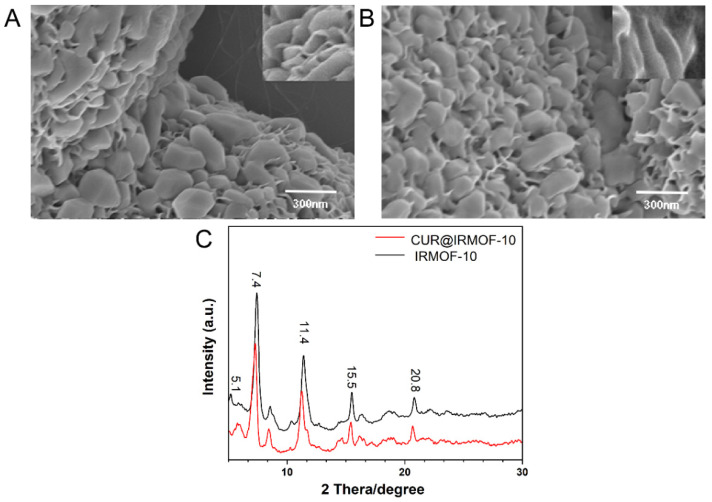
Characteristics of IRMOF-10 and CUR@IRMOF-10: (**A**) SEM image of IRMOF-10; (**B**) SEM image of CUR@IRMOF-10; (**C**) X-ray diffraction (XRD) analysis of IRMOF-10.

**Figure 4:**
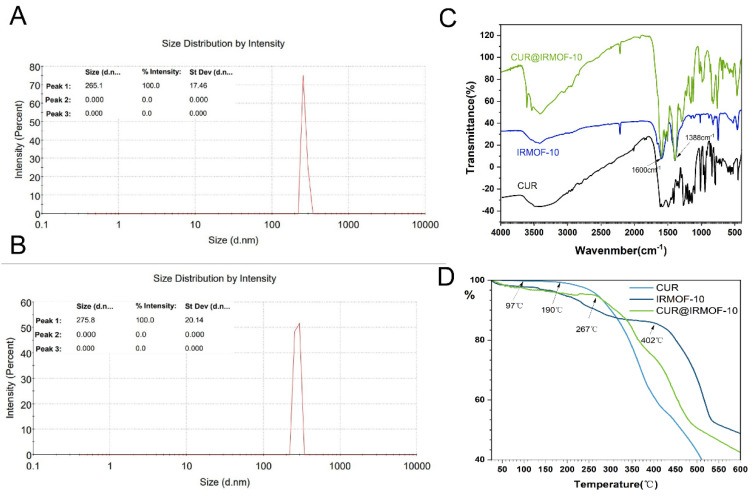
Particle size distribution of (**A**) IRMOF-10 and (**B**) CUR@IRMOF-10; (**C**) Fourier transform infrared spectrometer (FTIR) spectra of IRMOF-10 and CUR@IRMOF-10; (**D**) thermogravimetric (TG) analysis of IRMOF-10 and CUR@IRMOF-10.

The surface morphology of IRMOF-10 was observed with SEM (Figure 3A), revealing a solid polymer with nanoflake accumulation. The reason for this may be the slightly high sample concentration, which constituted the sample pile. XRD analyzed the crystallinity and grain size of IRMOF-10. As shown in Figure 3C, the IRMOF-10 crystallinity formed by the triethylamine method was good, and the characteristic diffraction peaks were 5.1, 7.4, 11.4, 15.5, and 20.8. The diffraction peaks were wide, indicating that the size of the nanomaterial was small. According to the particle size distribution diagram (Figure 4A), the particle size of IRMOF-10 was about 265.1 nm, and the particle size was concentrated, indicating that the size was uniform. To identify the structural composition and chemical groups of the nanoparticles, we used the infrared spectroscopy method, and the results are shown in Figure 4C. The structural frame vibration of IRMOF-10 at 900–1300 cm^−1^, the asymmetric telescopic vibration of the –COO key at 1600 cm^−1^, and the symmetric telescopic vibration at 1388 cm^−1^ are obvious. TG can measure the thermal stability of IRMOF-10 in nanoparticles. Figure 4D shows that IRMOF-10 had good thermal stability, and a slight weight loss occurring before 300 °C represented the weight loss of DMF and water molecules remaining in the framework structure. When the temperature gradually increased from 400 to 500 °C, the weight decreased significantly, indicating that the organic framework decomposed into carbon dioxide and water and continued to heat up; the weight no longer changed significantly, and the remainder was zinc oxide. The figure shows that the framework of IRMOF-10 was relatively stable before 400 °C, and the thermal stability was good. Our results showed that IRMOF-10 was synthesized successfully.

### 2.2. Drug Loading

The optimal process selected by the orthogonal test involved CUR being loaded for 24 h, an IRMOF-10/CUR ratio of 2:3, and a CUR concentration of 3 mg/mL. The verification process results showed that the drug loading was 63.96% (Tables S1–S4).

CUR@IRMOF-10 was characterized by SEM, XRD, DLS, FTIR, TG, and BET.

Through SEM observation (Figure 3B), the particle size of the material was seen to have increased after loading with CUR.

According to the XRD experiment (Figure 3C), the main diffraction peaks of CUR@IRMOF-10 shifted slightly, and the diffraction peaks increased.

The DLS experiment (Figures S1 and S2) showed that the particle size after loading with CUR was 275.8 nm (Figure 4B), which demonstrated no significant difference from IRMOF-10 (Figure 4A).

As shown in the FTIR diagram of CUR in Figure 4C, 3519 cm^−1^ was the O–H vibration of the phenolic group, 1599 cm^−1^ was the benzene ring stretching vibration, and 1509 cm^−1^ was the stretching vibration of C=O [4]. In the FTIR image of CUR@IRMOF-10 (Figure 4C), the characteristic peaks of curcumin are partially covered, and the characteristic peaks of IRMOF-10 are preserved. The two peaks at 1596 and 1502 cm^−1^ are consistent with the peaks generated by the stretching vibration of the benzene ring skeleton and the stretching vibration of C=O in CUR, proving that curcumin was adsorbed on the pores and surfaces of IRMOF-10. In addition, the following drug-release experiments also confirmed that CUR was indeed adsorbed by IRMOF-10 in a special binding manner. The release rate of IRMOF-10 was slower compared with other materials, probably due to some host–guest reactions between curcumin and IRMOF-10.

In the TG graph, the mass loss of CUR starts at 97 °C, decreases significantly at 190 °C, and becomes more pronounced after 245 °C, which is consistent with literature reports [25,26]. Both IRMOF-10 and CUR@IRMOF-10 have some weight loss before 300 °C due to the moisture and volatile impurities contained in them. Moreover, the weight of CUR@IRMOF-10 is significantly reduced at 267 °C, which indicates that the material can increase the thermal stability of CUR. This result indicates that the CUR was successfully loaded into IRMOF-10. However, the literature also describes a special binding mode between CUR and metal ions [27,28,29,30]. Therefore, in addition to encapsulation, there may also be some curcumin loaded on IRMOF-10 in a chelating manner with metal ions, explaining why the FTIR of CUR@IRMOF-10 retained some of the curcumin’s characteristic peaks.

**Figure 5:**
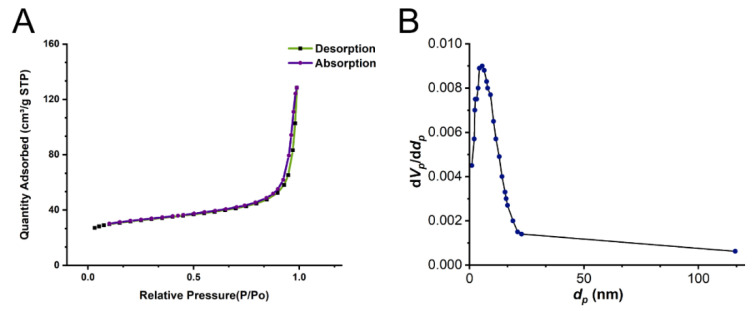
Brunauer–Emmett–Teller (BET) of IRMOF-10: (**A**) N_2_ adsorption and desorption isotherm of IRMOF-10; (**B**) pore size distribution of IRMOF-10.

In the BET experiment, the specific surface area of IRMOF-10 was 118.877 m^2^/g, the total pore volume was 0.199 cm^3^/g, and the pore size was about 5.56 nm. The N_2_ adsorption–desorption results for IRMOF-10 are shown in Figure 5A, and the pore size distribution is shown in Figure 5B. Our results showed that the synthesized IRMOF-10 had a nanoporous structure, which, together with the above results, proved that IRMOF-10 was suitable for the subsequent experiments.

### 2.3. In Vitro Release

**Figure 6:**
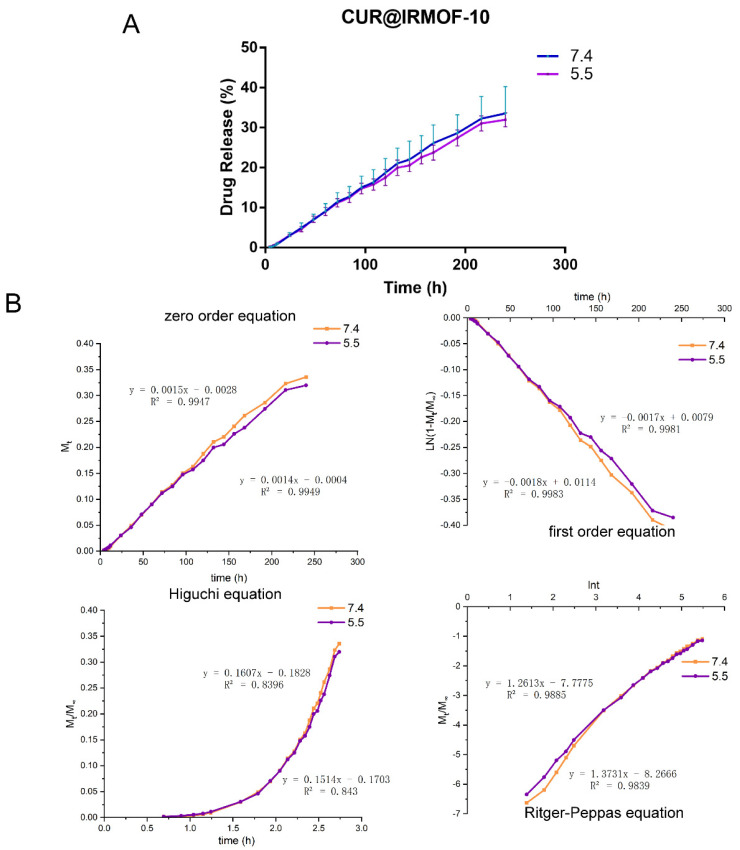
Release characteristics of CUR@IRMOF-10: (**A**) release curve of CUR@IRMOF-10 under different pH conditions (*n* = 3); (**B**) fitting curve of CUR@IRMOF-10 by different mathematical models under different pH values (5.5 and 7.4).

The in vitro release characteristics of CUR@IRMOF-10 were investigated under two pH (7.4 and 5.5) conditions. The results are shown in Figure 6A. Within two hours, there was almost no difference in the proportion of drugs released under the above two conditions. As time passed, the proportion of drugs released was slightly different. When the time was 210–240 h, the drug-release rate under the two pH conditions slowed down, and both were about 30% (33.58% and 31.86%, respectively). The release proportion of CUR@IRMOF-10 under the pH 7.4 condition was slightly higher. Our results showed that the in vitro release behavior of CUR@IRMOF-10 was little affected by pH.

We used the zero-order, first-order, Higuchi, and Ritger–Peppas equations to curve-fit the in vitro release results (Figure 6B). The kinetic characteristics of in vitro drug release were described using zero-level and first-level kinetic equations. It can be seen from Figure 5 that, at pH values of 7.4 and 5.5, CUR@IRMOF-10 conformed to the first-order release process, which is in line with the characteristics of sustained-release drugs. The release mechanism was analyzed using the Higuchi and Ritger–Peppas equations. The correlation coefficient of the Higuchi equation was less than 0.99, indicating that the release behavior of CUR@IRMOF-10 did not fit. For the MOFs, the drug diffused from the skeleton, and the material also dissolved, accelerating the release of the drug [23]. The Ritger–Pappas equation was used to judge the matrix dissolution and drug-diffusion behavior during drug release. Our results showed that the coefficient of lnt in this model was >0.89, indicating that the in vitro drug-release behavior of CUR@IRMOF-10 was mainly caused by the dissolution of IRMOF-10 [31].

### 2.4. In Vitro Study on the Safety of IRMOF-10

**Figure 7:**
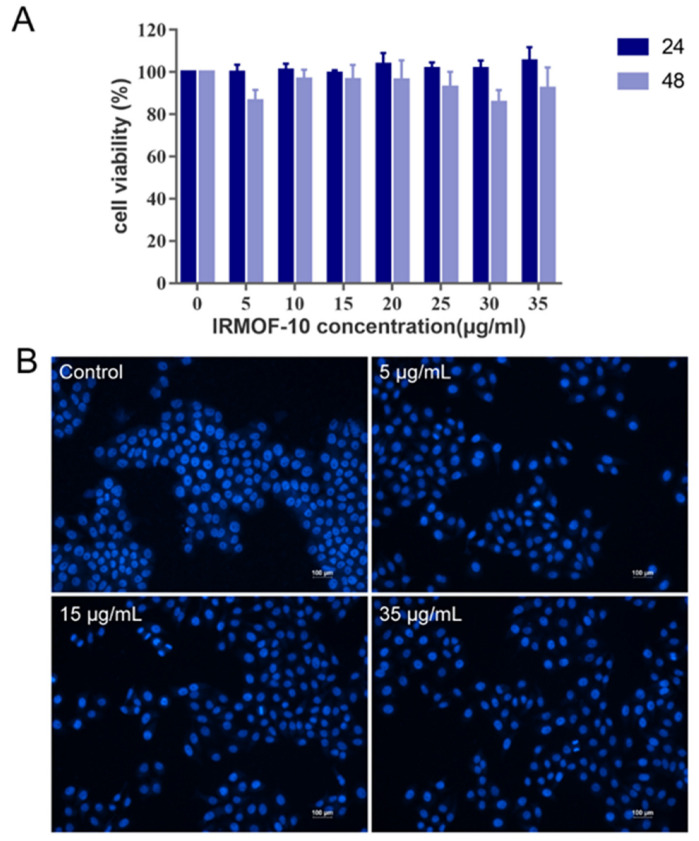
Safety of IRMOF-10: (**A**) MTT method to determine the safe concentration range of IRMOF-10 for HepG2 cells; (**B**) fluorescence micrographs of HepG2 cells stained with DAPI after 24 h of treatment with three concentrations of IRMOF-10.

**Figure 8:**
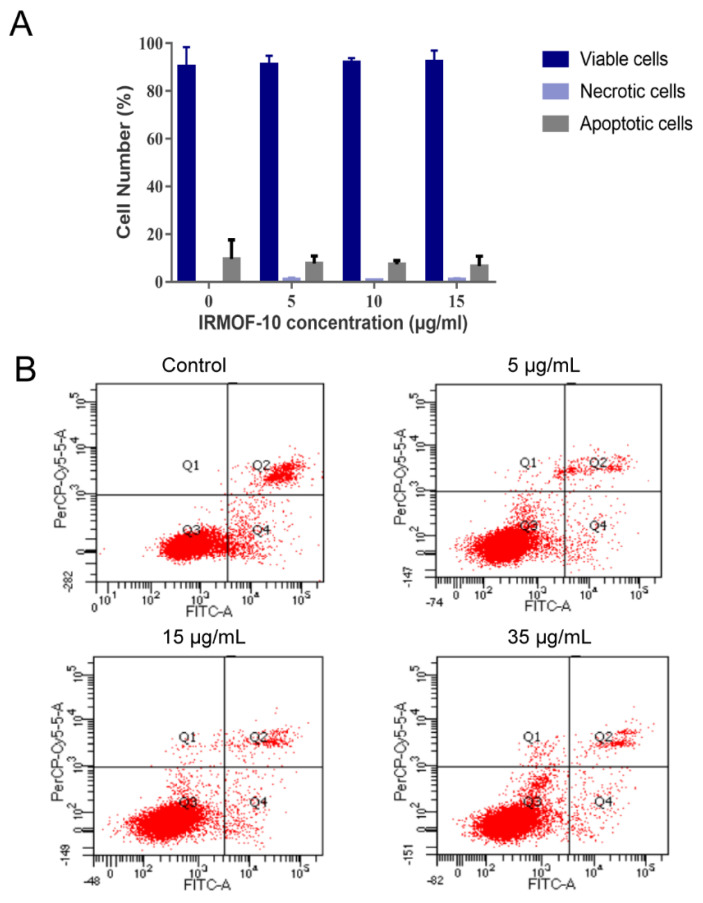
Safety of IRMOF-10: (**A**) proportion of viable, necrotic, and apoptotic HepG2 cells after incubation with different concentrations of IRMOF-10 for 24 h; (**B**) flow cytometry detection of apoptosis with FITC-annexin V/PI double staining.

Firstly, an MTT assay was used to detect the toxicity of different concentrations of IRMOF-10 in HepG2 cells. HepG2 cells were cultured for 24 and 48 h with different concentrations of IRMOF-10 (5, 10, 15, 20, 25, 30, and 35 μg/mL) and our results showed that the viability of the HepG2 cells was higher (Figure 7A), indicating that IRMOF-10 is not toxic to HepG2 cells at the experimental doses. We also observed the morphological changes of the HepG2 cell nucleus at different concentrations of IRMOF-10 (5, 15, and 35 μg/mL) after 24 h through 4′-6-diamidino-2-phenylindole (DAPI) staining. As shown in Figure 7B, during the retest dose range, the morphology of the HepG2 nucleus was good and without rupture, deformation, or other phenomena. In addition, we detected the effects of IRMOF-10 on apoptosis using the annexin V/PI method to further verify the safety of IRMOF-10 (Figure 8). Our experimental results showed that there was no significant difference in the proportion of living, necrotic, and apoptotic cells under different concentrations of IRMOF-10. Again, this finding proved our conclusion that IRMOF-10 is non-toxic to HepG2 cells at the experimental doses.

### 2.5. CUR@IRMOF-10 Induces Apoptosis of HepG2 Cells

#### 2.5.1. Apoptosis

**Figure 9:**
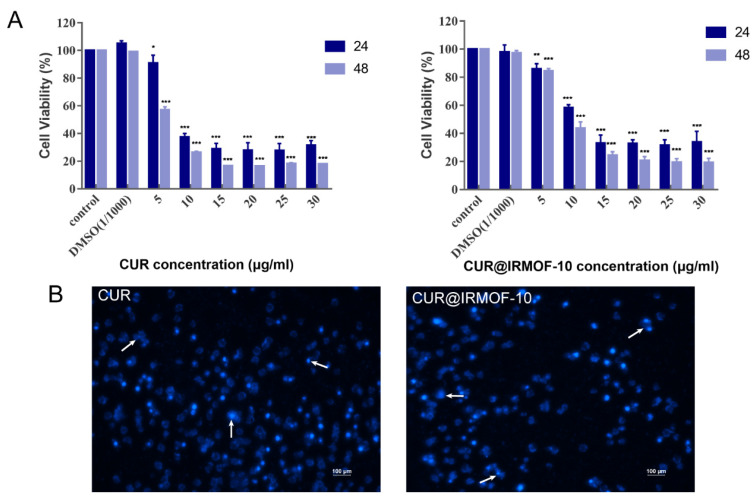
Toxicity of CUR and CUR@IRMOF-10 for HepG2 cells: (**A**) cell survival rate of HepG2 cells incubated with different concentrations of CUR and CUR@IRMOF-10 for 24 and 48 h—* *p* < 0.05, ** *p* < 0.01, and *** *p* < 0.001 vs. control; (**B**) fluorescence microscopic images of HepG2 cells stained with DAPI after 24 h treatment with CUR or CUR@IRMOF-10 (at IC_50_ value).

CUR induces the apoptosis of HepG2 cells [32]. As shown in the MTT experiment in Figure 9A, the low concentration (5 μg/mL) of CUR had little difference on the control group in terms of the cell survival rate at 24 h. However, the cell survival rate decreased significantly after HepG2 cells were treated at this concentration for 48 h. Clearly, curcumin is time-dependent in promoting the apoptosis of HepG2 cells. In addition, it is evident that the effects of CUR on promoting apoptosis are also concentration-dependent. When the concentration was increased to 10 μg/mL, the difference in the effect of each concentration was weakened at 24 and 48 h. When the concentration increased to 15 μg/mL, the intensity of apoptosis was no longer significantly different. The calculation of IC_50_ after 24 h of CUR acting on HepG2 cells was 9.213 μg/mL, as determined by using GraphPad Prism 7.0. As seen in the DAPI staining image (Figure 9B), curcumin was significantly toxic to HepG2 cells. When curcumin was used to culture HepG2 cells for 24 h, many cell ruptures, deformations, and nuclear pyknosis phenomena appeared, which is consistent with the literature [32]. HepG2 cells were co-cultured with CUR@IRMOF-10 (IC_50_) for 24 h and then stained with DAPI. As seen in Figure 9B, both CUR@IRMOF-10 and CUR can induce apoptosis of HepG2 cells.

**Figure 10:**
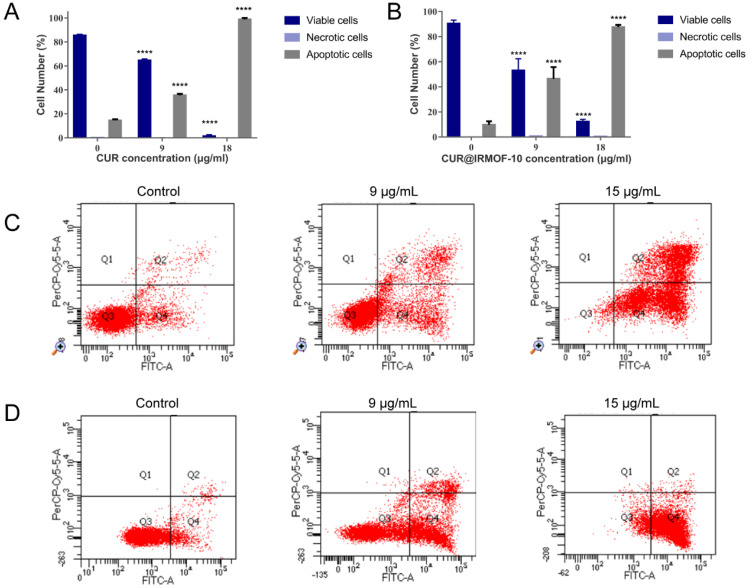
Statistical analysis of viable, necrotic, and apoptotic HepG2 cells with CUR (**A**) or CUR@IRMOF-10 (**B**)—**** *p* < 0.0001 vs. control. Apoptosis assays for HepG2 cells after treatment with CUR (**C**) or CUR@IRMOF-10 (**D**).

The flow cytometry experiment sought to prove this effect. Based on our experimental results, we decided to use two concentrations, 9 and 18 μg/mL, for the experiment. As shown in Figure 10, both CUR and CUR@IRMOF-10 could promote cell apoptosis. Different concentrations of CUR@ IRMOF-10 had significantly different effects on HepG2 cells. At high concentrations (18 μg/mL), at 24 h, CUR@IRMOF-10 promoted cell apoptosis more weakly than CUR (18 μg/mL). However, when the concentration was 9 μg/mL, at 24 h, the pro-apoptotic effect of CUR@ IRMOF-10 was stronger than that of CUR.

#### 2.5.2. Cellular Uptake

**Figure 11:**
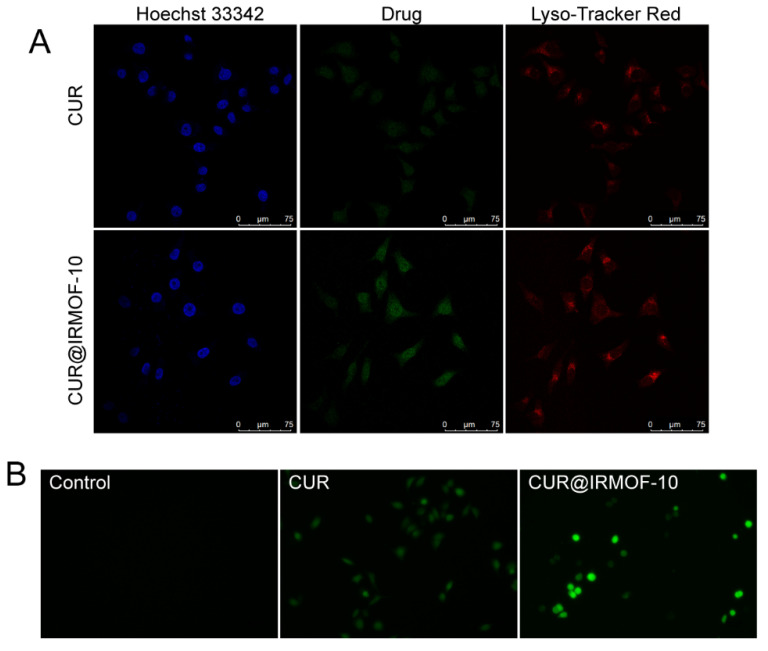
Cell uptake and ROS content determination: (**A**) confocal microscopy images of HepG2 cells after incubation with CUR for 30 min; (**B**) effect of CUR or CUR@IRMOF-10 on endogenous reactive oxygen species (ROS) level in HepG2 cells (administered concentration calculated based on IC_50_ of CUR).

Curcumin has fluorescent properties [33,34]. Using this feature, we could observe the CUR distribution in cells through CLSM and the influence of materials on the uptake of CUR by cells. HepG2 cells were co-cultured with CUR (9 μg/mL) or CUR@IRMOF-10 (9 μg/mL) for 30 min. Then, there were observed under a laser confocal microscope. Compared to the CUR group, CUR@IRMOF-10 showed stronger green fluorescence in the nucleus (Figure 11A), indicating that, in terms of intracellular uptake, CUR@IRMOF-10 has better nucleation characteristics than CUR.

#### 2.5.3. ROS

Oxidative stress is one of the most important factors that cause diseases [35]. The production of intracellular ROS is closely related to cell apoptosis [36]. CUR and CUR@IRMOF-10 were co-cultured with HepG2 cells for 24 h, and intracellular ROS was detected using an oxidation-sensitive fluorescent probe (DCFH-DA). The fluorescence of the CUR (9 μg/mL) and CUR@IRMOF-10 (9 μg/mL) groups was significantly stronger than the control group, and the fluorescence of CUR@IRMOF-10 at this concentration was significantly stronger than that of CUR (Figure 11B). This result was consistent with our experimental results.

#### 2.5.4. Mitochondrial Membrane Potential

**Figure 12:**
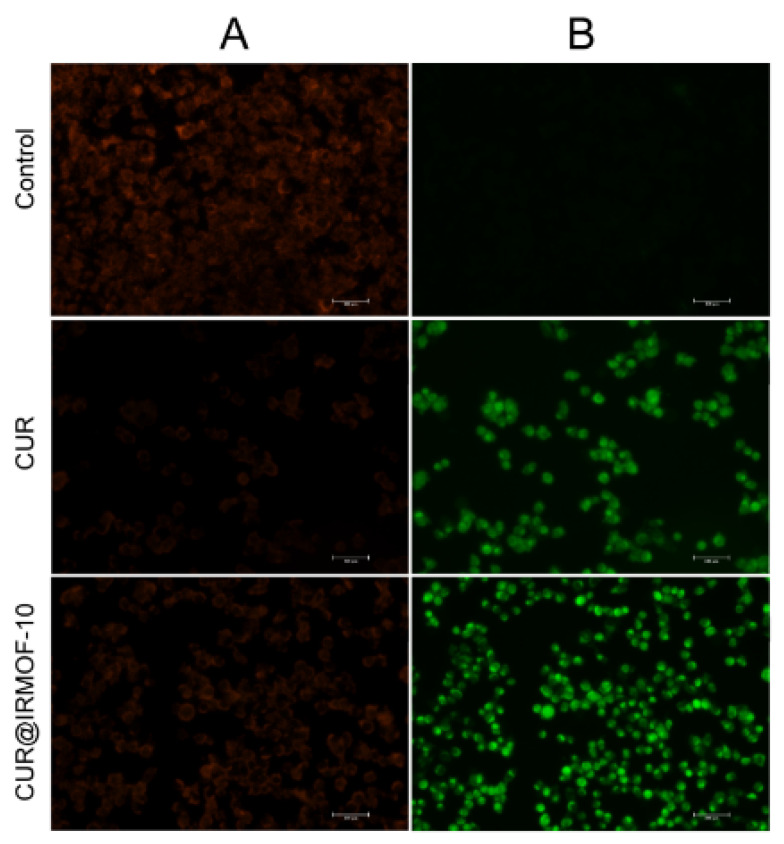
Effect of CUR and CUR@IRMOF-10 on the mitochondrial membrane potential of HepG2 cells. Red fluorescence represents the aggregate form of JC-1 (**A**); green fluorescence represents the monomeric form of JC-1 (**B**) (administered concentration calculated based on IC_50_ of CUR).

Studies have proven that ROS production is closely related to reducing the mitochondrial membrane potential (MMP) [36]. CUR and CUR@IRMOF-10 were co-cultured with liver cancer cells for 24 h, and the mitochondrial membrane potential (Δψm) of HepG2 cells in the control group and the treatment groups was measured using fluorescent, lipophilic, and cationic JC-1 probes (Beyotime, Shanghai, China). Our results showed that the green fluorescence intensity of the administration group with high reactive oxygen species was higher than for the control group, and the green fluorescence of CUR@IRMOF-10 was significantly stronger than that of CUR (Figure 12). This finding also agreed with our experimental results.

## 3. Discussion

In this study, IRMOF-10 was used as the carrier of the anti-liver cancer active ingredient CUR, and CUR@IRMOF-10, a nanoscale sustained-release drug delivery system, was successfully prepared. CUR@IRMOF-10 has a high drug-loading capacity, good biocompatibility, and a slow-release effect, while retaining curcumin’s anticancer effects. Therefore, the material is safer than curcumin. Yaghi et al. [15] first used the solvothermal method to synthesize the IRMOF series. In previous studies, IRMOF-10 was also mainly synthesized by the solvothermal method [37,38,39]. Yet, in this paper, we used the direct addition method of triethylamine to synthesize IRMOF-10, an optimized version of the method used by Inés Gutiérrez [40]. Compared to the solvothermal method, this method can be performed at room temperature, which is safer and more convenient, and can rapidly synthesize MOFs in a short time [4]. To date, many materials have been used as carrier materials for curcumin, such as CUR-loaded solid lipid nanoparticles (37% ± 2.5%) [41], CUR-loaded chitosan nanoparticles (18%) [42], CUR-loaded mesoporous silica nanoparticles (MSNs), and CUR-loaded amine-functionalized MSNs (7.9%, 24.4%) [43]. The common drug-loading methods for MOFs include the solvent adsorption method [44], covalent crosslinking method [45], and an insitu growth method [46]. IRMOF-10 has great advantages in terms of drug loading, and in this study, the solvent adsorption method was applied to it, achieving drug loading of CUR@IRMOF-10 that reached 63.96%.

IRMOF-10 and CUR@IRMOF-10 were characterized by SEM and other experiments, and the results proved that CUR was successfully loaded into IRMOF-10. CUR@IRMOF-10 can improve the thermal stability of CUR. As CUR@IRMOF-10 is 264 °C and CUR weight loss occurs at 190 °C(Figure 4D), the rate of weight loss is slower(Figure S3). The special binding mode between curcumin and the carrier mentioned above may be related to the structure of CUR. The 1,3-diketone structure of CUR could be automatically converted into the keto-enol tautomeric form; the keto-enol is more stable and easily chelates metal ions such as Zn^2+^ and Cu^2+^ [47,48,49]. In the in vitro drug-release experiment, the release of CUR was very slow; only about 30% of the drug was released after 10 days, which is consistent with the literature [50]. This enol-like combination may be the cause of high drug loading and slow drug release.

We investigated the biological safety of IRMOF-10 and the apoptosis-promoting effect of CUR and CUR@IRMOF-10 via an MTT assay, DAPI staining, and annexin V/PI. Our results showed that IRMOF-10 in this experimental concentration has no toxicity, proving that IRMOF-10 is a good nanocarrier candidate for drug delivery in human bodies. Moreover, both CUR and CUR@IRMOF-10 induced apoptosis in HepG2 cells. The MTT and annexin V/PI experiments proved that CUR@IRMOF-10 helped to enhance the antitumor effect of CUR. At the same time, the IC50 of CUR@IRMOF-10 was lower than that of CUR. At the same concentration, the apoptosis rate of the CUR@IRMOF-10 group was higher than that of the CUR group. Combined with the uptake experiment, it can be seen that CUR@IRMOF-10 more easily enters the nucleus than CUR. So, it can achieve the increase in reactive oxygen species and decrease in mitochondrial membrane potential faster, meaning it can function better. However, at a high concentration (18 μg/mL), CUR@IRMOF-10 has a slightly lower efficacy than free CUR, which may be related to the slow-release characteristics of IRMOF-10.

Apoptosis is closely related to oxidative stress. Previous studies have shown that ROS can trigger apoptosis by activating the p38 MAPK signaling pathway [51], enhancing endoplasmic reticulum (ER) stress [52], upregulating p53, and activating the mitochondrial apoptosis pathway [36]. Through the ROS experiment, we found that the ability of CUR@IRMOF-10 to induce ROS was stronger than that of CUR. Many studies have proven that ROS are closely related to mitochondria. ROS can directly damage the integrity of the mitochondrial membrane by hyperpolarizing the mitochondrial membrane and releasing cytochrome c into the cytoplasm [53,54]. In addition, ROS can also damage mitochondrial DNA, which is related to the respiratory chain, thereby aggravating the production of ROS [55]. On the other hand, a large amount of ROS may also cause nuclear DNA damage [56], upregulate the expression of p53, block the cell cycle in the S phase, and activate the mitochondrial-dependent apoptotic pathway [36]. Furthermore, the mitochondrial-dependent apoptosis pathway is an important way to achieve anti-tumor effects [57]. For example, BH3-mimetics, an anticancer drug, achieves anti-tumor effects through the mitochondrial-dependent death pathway [58,59]. After the mitochondria are damaged, the released cytochrome c forms apoptotic bodies with apoptotic peptidase activating factor 1 (APAF-1) and dATP in the cytoplasm. Apoptotic bodies recruit and activate procaspase-9, which activates other downstream caspases to initiate apoptosis [60,61]. MMP can reflect mitochondrial dysfunction, and the decrease in mitochondrial membrane potential is a landmark event in the early stages of apoptosis. The results of our study prove that CUR@IRMOF-10 has a stronger effect on promoting the entry of the CUR into the nucleus, generating ROS, and reducing the mitochondrial membrane potential than CUR, suggesting that IRMOF-10 enhances the effect of CUR in terms of promoting cell apoptosis.

## 4. Materials and Methods

### 4.1. Reagents

Zinc nitrate hexahydrate (Zn(NO_3_)_2_·6H_2_O) was purchased from Shanghai Aladdin Biochemical Technology Co., Ltd. (Shanghai, China); triethylamine (TEA) and 4′,4-biphthalic acid (4,4′-BPDC) were bought from the Tianjin Guangfu Fine Chemical Research Institute (Tianjin, China); *N*,*N*-dimethylformamide (DMF) and trichloromethane (CHCl_3_) were obtained from the Beijing Chemical Factory (Beijing, China); methanol (chromatographic reagent grade) was obtained from Thermo Fisher Scientific (Shanghai, China); curcumin (CUR)( Figures S4–S7) was purchased from Shanghai Yuanye Bio-Technology Co., Ltd. (LOT:R12A10S85604, 98%); dimethyl sulfoxide (DMSO), high-glucose Dulbecco’s modified Eagle’s medium (DMEM), PBS, and a penicillin–streptomycin mixture were purchased from Solarbio (Beijing, China); fetal bovine serum (FBS) was acquired from Corning; 3-(4,5-dimethylthiazol-2-yl)-2,5-dipheny-ltetrazolium bromide (MTT) was acquired from Beijing BioDee Biotechnology Co., Ltd. (Beijing, China); 4′,6-diamidino-2-phenylindole (DAPI), an Annexin V-FITC Apoptosis Detection Kit, Lyso-Tracker Red Kit, ROS assay kit, and mitochondrial membrane potential assay kit with JC-1 were purchased from Shanghai Beyotime Biotechnology Co., Ltd. (Shanghai, China); HepG2 cells were purchased from Guangzhou Jeniobio Biotechnology (Beijing, China).

### 4.2. Preparation of IRMOF-10 and Drug-Loading

#### 4.2.1. Preparation of IRMOF-10

We adopted the triethylamine direct addition method, whereby 4′,4-biphthalic acid (0.24272 g) and zinc nitrate hexahydrate (0.61074 g) were placed into a 150 mL conical bottle, and 100 mL of DMF was added. The bottle was sealed to prevent water vapor from entering and treated ultrasonically for 10 min to completely dissolve the 4′,4-biphthalic acid and zinc nitrate hexahydrate. Then, the mixture was stirred at a speed of 800 rpm using a magnetic agitator. At this time, 11 mL of TEA was added quickly, the container was sealed, and stirring was continued for 2 h at 800 rpm and at room temperature.

After completing this, the reaction solution was collected by centrifugation. The precipitate was washed twice with DMF and trichloromethane, respectively, and soaked in 30 mL of trichloromethane for three days, during which time the trichloromethane was changed once a day. Then, the precipitate was placed into a vacuum drying oven for 12 h, removed, and stored in a dryer.

#### 4.2.2. Drug Loading

We used the solvent adsorption method to load CUR into IRMOF-10. An orthogonal experiment was performed to determine the optimal drug-loading process. We selected three factors for the orthogonal experiment: the weight ratio of IRMOFs and CUR (3:2, 1:1, 2:3), CUR loading time (6, 12, and 24 h), and CUR concentration (1, 2, and 3 mg/mL). The CUR concentration in the supernatant after CUR loading was determined by using a Thermo Scientific UltiMate 3000 HPLC (Waltham, MA, USA). The drug-loading calculation was

(1) $$drug\;loading=\frac{weight\;of\;drug\;in\;NPs}{weight\;of\;NPs}\times 100\%$$

### 4.3. In Vitro Release Study

For the in vitro release, we used the dialysis method to obtain the CUR@IRMOF-10 profiles. Typically, CUR@IRMOF-10 was placed into dialysis bags and immersed in phosphate-buffered saline (PBS) at pH 7.4 and 5.5, while stirring for 100 rpm at 37 °C simultaneously. Then, the amount of CUR released solution was recorded at a set time interval, and the same amount of fresh PBS was added. HPLC determined the drug release. The corrected concentration and the proportion of CUR released were expressed as

(2) $$C_{c}=C_{t}+\frac{v}{V}\overset{t-1}{\underset{0}{\sum }}C_{t},$$

(3) $$Drug\;release=\frac{M_{R}}{M_{L}}\times 100\%$$

In Equation (2), *C_c_* stands for the adjusted concentration of CUR at time *t*, *C_t_* is the measured concentration at *t*, *v* is the volume of the derived samples, and *V* is the volume of release solution. *M_R_* and *M_L_* are the amounts of released and loaded drugs (Equation (3)), respectively.

### 4.4. Characterization

The morphology and composition of the surface ultrastructure of IRMOF-10 and CUR@IRMOF-10 were observed by SEM images, which were taken using a high-performance field-emission scanning electron microscope (Zeiss, Oberkochen, Germany). Gold spraying was required for 10 min before testing. An X-ray diffractometer (XRD) was used to observe the crystal shape and size of IRMOF-10 and CUR@IRMOF-10 at 5°–30° using Cu-Kα (λ = 1.541 nm) radiation at 40 Kv and 40 mA via an X-ray powder diffraction instrument (Nippon Science Group Corporation, Tokyo, Japan). The particle size distribution was recorded by dynamic light scattering (DLS) using a Malvern Nano Zetasizer S90 (Malvern, UK). The Fourier-transform infrared (FTIR) spectra for IRMOF-10 and CUR@IRMOF-10 were determined using a Fourier-Transform Infrared Spectrometer (Thermo Scientific, Waltham, MA, USA) from 400 to 4000 cm^−1^. The relationship between the weight loss and temperature was recorded by DTG-60/60 simultaneous thermogravimetry/differential thermal analyzers (SHIMADZU, Kyoto, Japan), and a thermogravimetric (TG) map was obtained. The TG spectrum determined the thermal stability of the MOFs. The samples were heated in aluminum pans from 30 to 600 °C with a heating rate of 10 °C/min under N_2_ flow. The surface area and pore size of IRMOF-10 were measured using a Belsorp-max BET Sorptometer (Bel Japan Inc., Tokyo, Japan) at 77 K after being degassed at 150 °C for 12 h.

### 4.5. Cell Cultures and Treatments

The HepG2 cells were cultured in high-glucose Dulbecco’s modified Eagle’s medium (DMEM) supplemented with 10% fetal bovine serum (FBS) and 1% penicillin–streptomycin mixture at 37 °C, with 5% CO_2_, and in a humid atmosphere for two to three days. The IRMOF-10, free CUR, and CUR@IRMOF-10 were dissolved in dimethyl sulfoxide (DMSO) and then diluted with a fresh medium.

### 4.6. Cell Viability Assay

An MTT assay was used to analyze the viability of cells after administration. Cells were seeded on 96-well plates at a density of 5 × 10^3^ per well. The cells were incubated overnight in a cell culture incubator and administered after adherence. Then, the HepG2 cells were treated with IRMOF-10 (3, 6, 9, 12, 15, and 18 μg/mL), free CUR (5, 10, 15, 20, 25, and 30 μg/mL), and CUR@IRMOF-10 (3, 6, 9, 12, 15, and 18 μg/mL) for 24 h or 48 h. Afterward, an MTT solution (5 mg/mL) was added to the wells, and then incubated for 4 h. Finally, the supernatant was aspirated, and 150 μL DMSO was added to dissolve the crystals, shaken with a shaker for 10 min to dissolve the crystals completely. We used a BMG SPECTROstar Nano high-throughput microplate UV spectrophotometer (Ortenberg, Germany), after shaking for 10 min, to measure the absorbance of the cells at 490 nm. We conducted this in triplicate, in parallel, to calculate the cell survival rate.

### 4.7. Nuclear Morphology Assay Using DAPI Staining

The cells were seeded in six-hole plates with a density of 4 × 10^5^ cells per well, incubated overnight, and then treated with different concentrations of IRMOF-10, free CUR, and CUR@IRMOF-10 for 24 h before staining. Afterward, the cells were washed twice with PBS and fixed with 4% tissue fixative at room temperature for 10 min. The next step was to stain the cells with DAPI solution at room temperature for five minutes in the dark. Finally, they were washed twice with PBS for three minutes each time. The changes in stained cell nuclei were observed using a Nikon Research Inverted Microscope ECLIPSE Ts2R (Tokyo, Japan).

### 4.8. Analysis of Apoptosis by Flow Cytometry

According to the instructions, the apoptosis of HepG2 cells was detected by an Annexin V-FITC/PI apoptosis detection kit. HepG2 cells (4 × 10^5^ cells/well) were inoculated in a six-well plate and cultured overnight. Then, free CUR and CUR@IRMOF-10 with different concentrations were incubated for 24 h. Subsequently, the HepG2 cells were processed according to the instructions. Afterward, the cells were suspended in 195 μL annexin V binding buffer, stained with 5 μL FITC-labeled annexin V and 10 μL polyimide, and incubated in the dark for 20 min. Then, the cells were immediately analyzed using a BD FACSCanto II flow cytometer (Franklin Lakes, NJ, USA).

### 4.9. CLSM for Cell Uptake

HepG2 cells were seeded in a laser confocal dish at a density of 2 × 10^5^ per well and cultured overnight. CUR and CUR@IRMOF-10 were added to each well. After culturing for 2 h, the mixture was washed three times with PBS; LysoTracker Red was added and incubated for 30 min. Afterward, the mixture was washed with PBS three times, fixed with 4% paraformaldehyde for 10 min, washed twice with PBS, and DAPI solution was added into the laser confocal dish and incubated for 20 min. Finally, the HepG2 cells were washed with PBS three times and observed using a confocal laser scanning microscope (Leica, Germany).

### 4.10. Intracellular ROS Detection

The ROS level of HepG2 cells was measured by a 2,7-dichlorofluorescein diacetate (DCFH-DA) assay. Firstly, the HepG2 cells were inoculated in a six-well plate, at a density of 2 × 10^5^ cells/well, and incubated overnight. Then, CUR and CUR@IRMOF-10 were used to stimulate the cells and culture them for 24 h. The medium was removed, and 1 mL of diluted DCFH-DA was added. The mixture was incubated at 37 °C for 20 min. The cells were washed three times with a serum-free cell culture medium to completely remove non-cellular DCFH-DA. Finally, a Nikon Research Inverted Microscope ECLIPSE Ts2R (Tokyo, Japan) was used to observe the cells.

### 4.11. Mitochondrial Membrane Potential (MMP)

Firstly, HepG2 cells were cultured on a 12-well plate at a density of 2 × 10^5^ cells per well for 10 h at 37 °C. Secondly, IRMOF-10, CUR, and CUR@IRMOF-10 were added to each well and cultured for 24 h. Afterward, they were washed with PBS, and the prepared JC-1 working solution was added and incubated for 30 min. Then, the cells were washed twice with ice-cold JC-1 buffer (1×). Finally, 1 mL of DMEM was added and observed under a Nikon Research Inverted Microscope ECLIPSE Ts2R (Tokyo, Japan), with pictures captured randomly.

## 5. Conclusions

In this study, IRMOF-10 was successfully synthesized and loaded with curcumin, an anticancer model component. Our results showed that IRMOF-10 had a good biocompatibility, a high CUR loading, and a slow-release effect. It thus represents an excellent drug delivery nanomaterial. In vitro cell experiments, MTT, DAPI staining, and apoptosis indicated that CUR@IRMOF-10 enhances apoptosis in HepG2 cells. Cell uptake, ROS, and MMP experiments indicated that the pro-apoptotic effects of CUR@IRMOF-10 may be related to the activation of the ROS-mediated mitochondrial apoptosis pathway.

## Data Availability

Not applicable.

## Supplementary Materials

The following supporting information can be downloaded at: https://www.mdpi.com/article/10.3390/molecules27123940/s1, Table S1: Orthogonal test factors; Table S2: Orthogonal test results; Table S3: Analysis of variance for the orthogonal test results; Table S4: Validation experiment results under optimum conditions; Figure S1: Size distribution report for IRMOF-10; Figure S2: Size distribution report for CUR@IRMOF-10; Figure S3: Differential thermogravimetric curve (DTG) of CUR (A) and CUR@IRMOF-10 (B); Figure S4: High Performance Liquid Chromatography (HPLC) of CUR; Figure S5: Thin Layer Chromatography (TLC) of CUR in Visible(A) and UV Light (365 nm) (B); Figure S6: ^1^H NMR and ^13^C NMR spectra of CUR; Figure S7: ^1^H NMR and ^13^C NMR spectra of bisdemethoxycurcumin and demethoxycurcumin.

## Sample Availability

Samples of the IRMOF-10 and CUR@IRMOF-10 are available from the authors.
