# Supplementary material for: Preparation, Characterization, and In Vitro Release of Curcumin-Loaded IRMOF-10 Nanoparticles and Investigation of Their Pro-Apoptotic Effects on Human Hepatoma HepG2 Cells
Source: Molecules. 2022 Jun 20;27(12):3940. doi: 10.3390/molecules27123940 (PMC9228752; doi:10.3390/molecules27123940)
Supplement: Supplementary file 1 [file molecules-27-03940-s001.zip › molecules-1693467-supplementary.pdf]

Table S1. Orthogonal tests factors.

Level	Factor		
	A<br>Time	B<br>IRMOF-10/CUR	C<br>CUR concentration
1	6h	3:2	1mg/ml
2	12h	1:1	2mg/ml
3	24h	2:3	3mg/ml

Table S2. Orthogonal test results

Group	A	B	C	Drug loading/%
1	2	1	3	41.01%
2	2	3	2	61.67%
3	1	3	3	65.96%
4	3	3	1	63.29%
5	1	1	1	10.01%
6	2	2	1	43.67%
7	3	1	2	41.30%
8	3	2	3	51.94%
9	1	2	2	55.85%
K1	43.940	30.773	38.990	
K2	48.783	50.487	52.940	
K3	52.177	63.640	52.970	
R	8.237	32.867	13.980	

Table S3. Analysis of variance for the orthogonal test results

Source of Variance	SS	df	variance	F	P
A(Time)	102.815	2	51.408	0.427	0.701
B(IRMOF-10/CUR)	1641.843	2	820.922	6.824	0.128
C(CUR concentration)	390.044	2	195.022	1.621	0.382
Error	240.587	2	120.294		

PS: $P<0.05$ means the difference is significant, $P<0.01$ means the difference is extremely significant

Results: Taking the drug loading as the inspection index, the results of the analysis of variance showed that B (IRMOF-10/CUR) had a significant difference on the experimental results, and A (Time) and C (CUR concentration) had no significant effects. The influence of various factors on the experimental results is B>C>A. According to the intuitive analysis of the orthogonal table, the optimal drug loading conditions are: $A_3B_3C_3$, that is, drug loading for 24 h, IRMOF-10/CUR is 2:3, and CUR concentration is 3 mg/mL.

Table S4. Validation experiment results under the optimum conditions

Number	DLC（%）	Average Value（%）	RSD（%）
1	62.93	63.96	1.15
2	64.67		
3	64.16		
4	64.10		

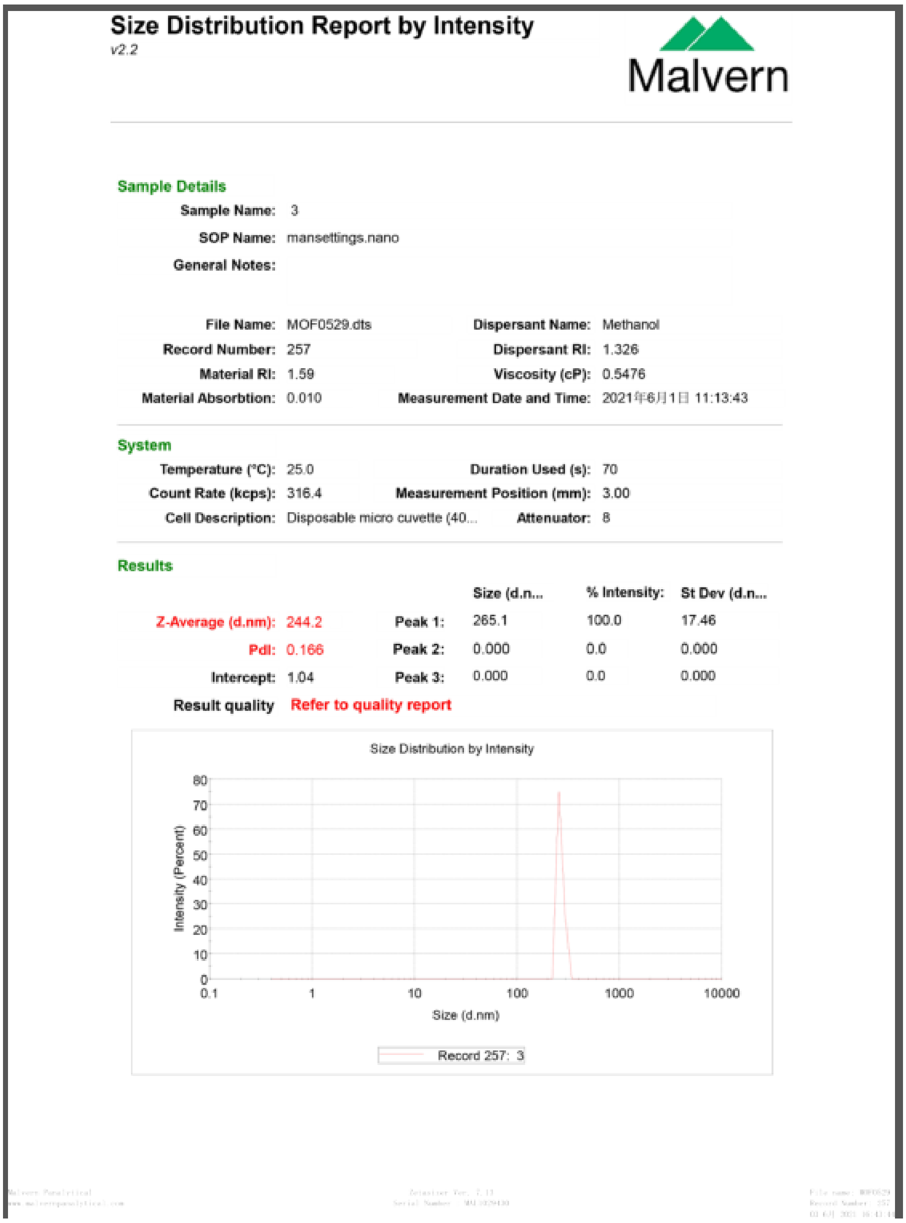

Figure S1: Size distribution report of IRMOF-10.

Size Distribution Report by Intensity

v2.2

Malvern

Sample Details

Sample Name:	1
SOP Name:	mansettings.nano
General Notes:	

File Name:	MOF0529.dts	Dispersant Name:	Methanol
Record Number:	288	Dispersant RI:	1.326
Material RI:	1.59	Viscosity (cP):	0.5476
Material Absorbtion:	0.010	Measurement Date and Time:	2021年6月1日 17:07:13

System

Temperature (°C):	25.0	Duration Used (s):	70
Count Rate (kcps):	206.6	Measurement Position (mm):	3.00
Cell Description:	Disposable micro cuvette (40...	Attenuator:	8

Results

			Size (d.n...	% Intensity:	St Dev (d.n...
Z-Average (d.nm):	255.9	Peak 1:	275.8	100.0	20.14
PdI:	0.243	Peak 2:	0.000	0.0	0.000
Intercept:	1.29	Peak 3:	0.000	0.0	0.000

Result quality Refer to quality report

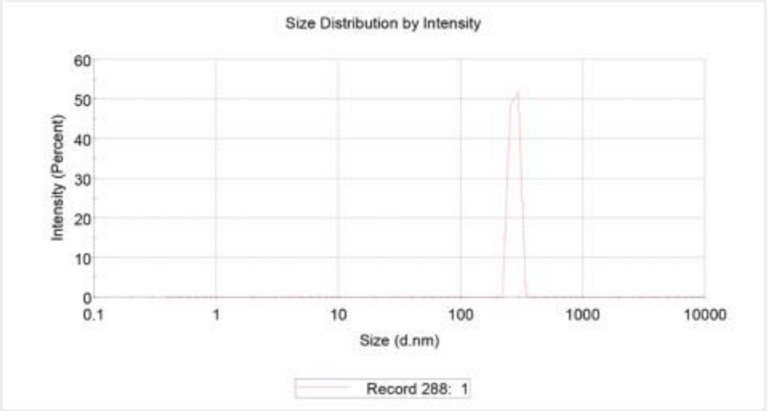

Figure S2: Size distribution report of CUR@IRMOF-10.

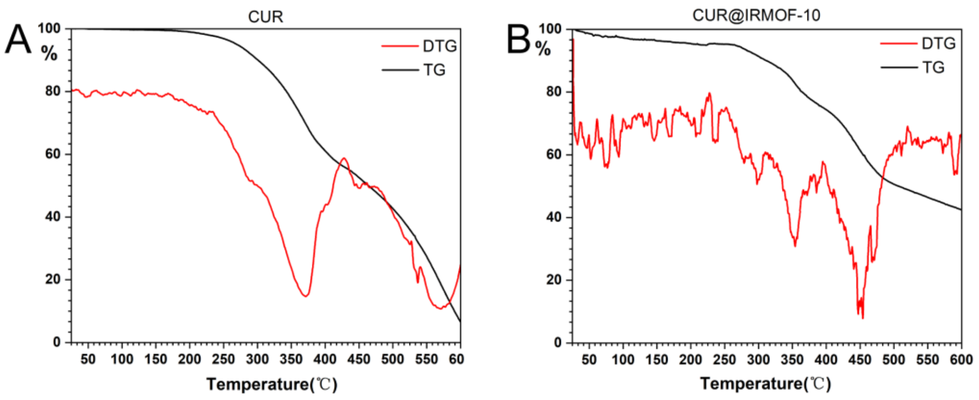

Figure S3: Differential Thermogravimetric Curve (DTG) of CUR(A) and CUR@IRMOF-10(B).

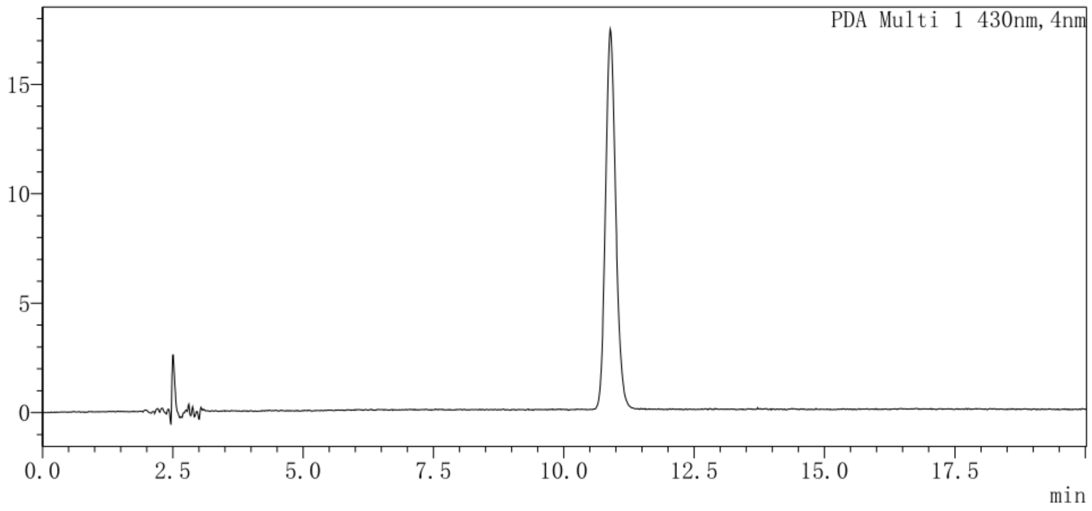

Figure S4: High Performance Liquid Chromatography (HPLC) of CUR

Method: Using a reversed phase column, Agilent ZORBAX SB-C18 (250mm×4.6mm, 5μm). The liquid chromatography system was LC-20A high performance liquid chromatograph equipped with a DAD diode array detector. Acetonitrile-4% glacial acetic acid solution (48:52) is used as mobile phase. The flow rate was constant at 1 mL/min, the injection volume was 10 μL, and samples were maintained at 25 ℃. Detection wavelength was 430 nm.

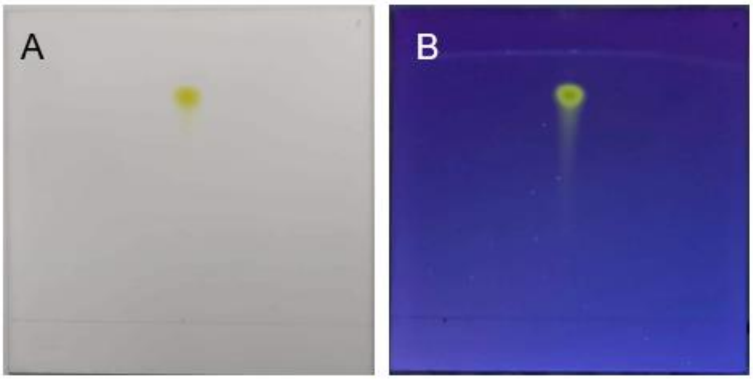

Figure S5: Thin Layer Chromatography (TLC) of CUR in Visible(A) and UV Light(365 nm)(B)

Method: Apply 4 μL of CUR solution (0.5 mg/mL) on a silica gel G thin-layer plate, and use chloroform:methanol:formic acid (96:4:0.7) as the mobile phase. The thin-layer plate is placed under sunlight and ultraviolet light(365 nm). respectively.

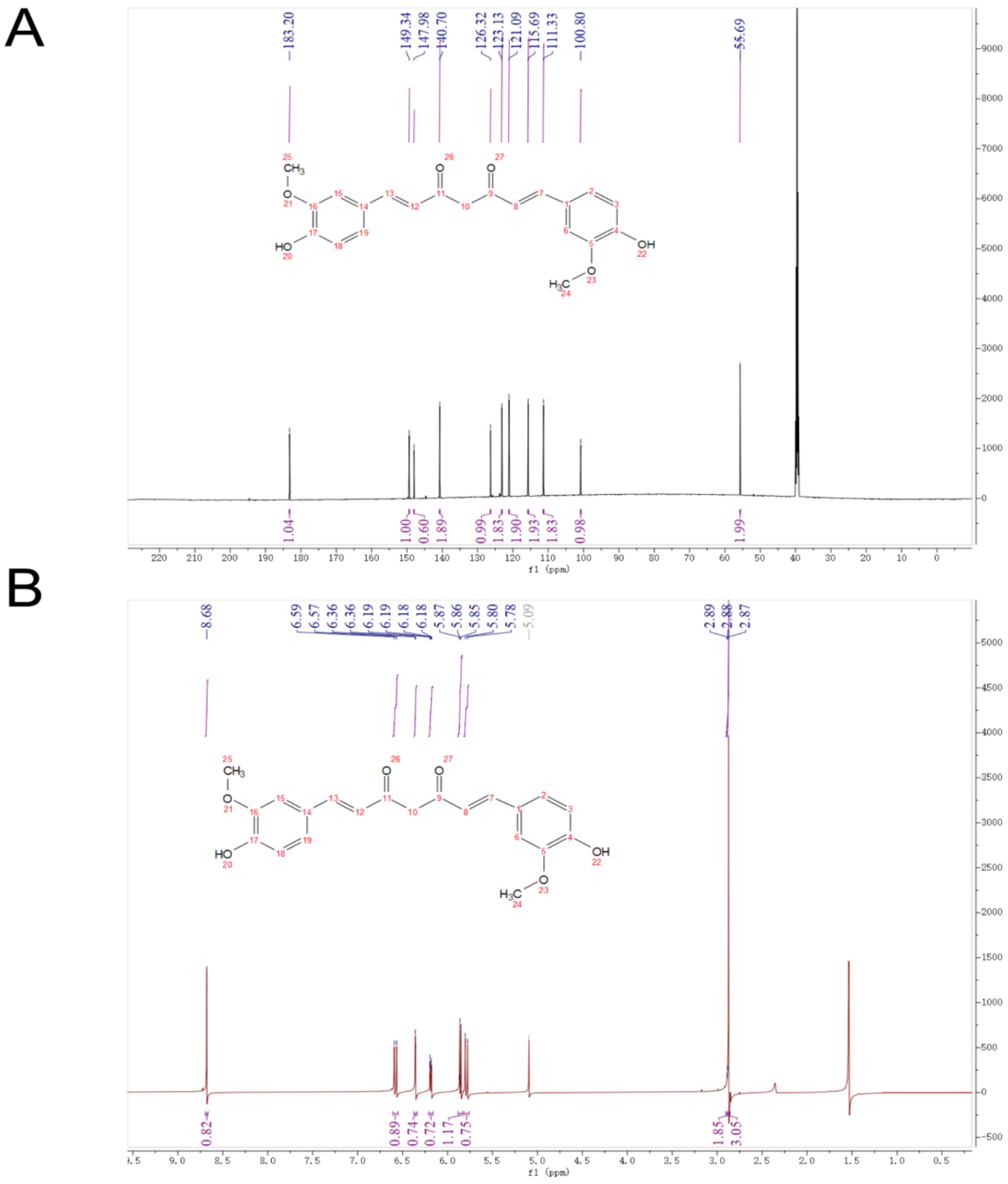

Figure S6: ^{1}H NMR and ^{13}C NMR spectra of CUR

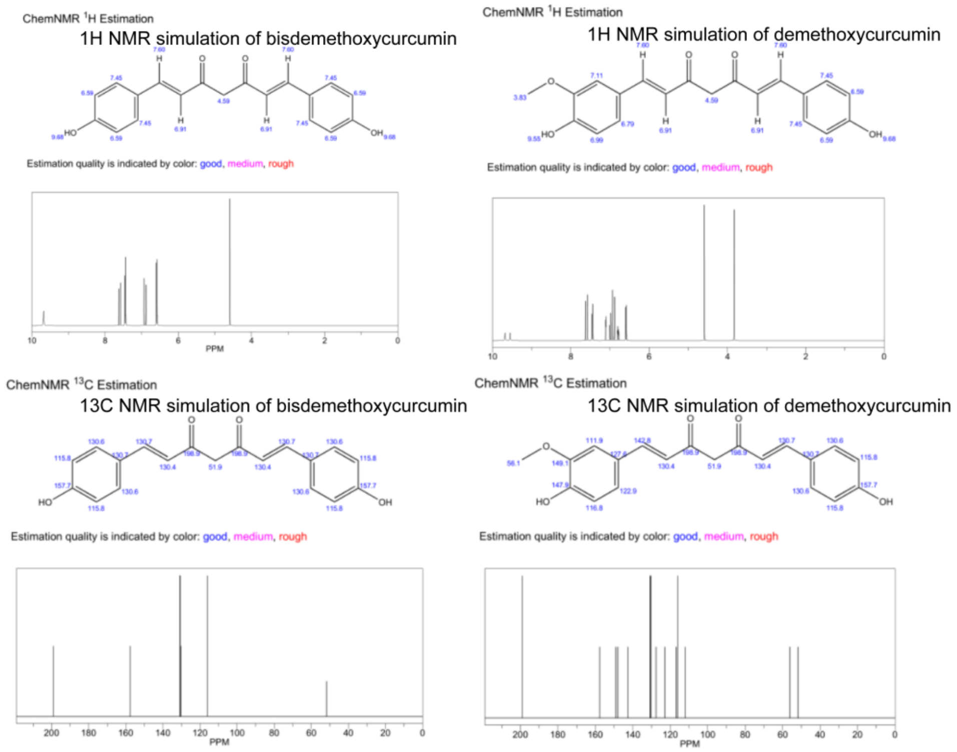

Figure S7: ^{1}H NMR and ^{13}C NMR spectra of bisdemethoxycurcumin and demethoxycurcumin
